# Effect of exercise on fluoride metabolism in adult humans: a pilot study

**DOI:** 10.1038/srep16905

**Published:** 2015-11-19

**Authors:** Fatemeh V. Zohoori, Alison Innerd, Liane B. Azevedo, Gary M. Whitford, Anne Maguire

**Affiliations:** 1 Health and Social Care Institute, Teesside University, Middlesbrough, UK; 2 School of Social Sciences, Business & Law, Teesside University, Middlesbrough, UK; 3 Department of Oral Biology, College of Dental Medicine, Georgia Regents University, Augusta, US; 4 Centre for Oral Health Research, School of Dental Sciences, Newcastle University, Newcastle-upon-Tyne, UK

## Abstract

An understanding of all aspects of fluoride metabolism is critical to identify its biological effects and avoid fluoride toxicity in humans. Fluoride metabolism and subsequently its body retention may be affected by physiological responses to acute exercise. This pilot study investigated the effect of exercise on plasma fluoride concentration, urinary fluoride excretion and fluoride renal clearance following no exercise and three exercise intensity conditions in nine healthy adults after taking a 1-mg Fluoride tablet. After no, light, moderate and vigorous exercise, respectively, the mean (SD) baseline-adjusted i) plasma fluoride concentration was 9.6(6.3), 11.4(6.3), 15.6(7.7) and 14.9(10.0) ng/ml; ii) rate of urinary fluoride excretion over 0–8 h was 46(15), 44(22), 34(17) and 36(17) μg/h; and iii) rate of fluoride renal clearance was 26.5(9.0), 27.2(30.4), 13.1(20.4) and 18.3(34.9) ml/min. The observed trend of a rise in plasma fluoride concentration and decline in rate of fluoride renal clearance with increasing exercise intensity needs to be investigated in a larger trial. This study, which provides the first data on the effect of exercise with different intensities on fluoride metabolism in humans, informs sample size planning for any subsequent definitive trial, by providing a robust estimate of the variability of the effect.

Fluoride (F) is a trace element which is naturally present in drinking water and all foods and drinks, especially tea and seafoods, at varying concentrations. Following absorption from the gastrointestinal tract, F is rapidly integrated into calcified tissues which contain 99% of body F. F status is of nutritional and public health significance and the American Dietetic Association^1^ has confirmed F as an important element for achieving and maintaining oral and bone health.

A major decline in the prevalence and severity of dental caries has been seen in many countries worldwide as a result of appropriate exposure to F in different forms added to drinking water, salt or milk. In the UK, more than 10% of the population receives fluoridated water at a concentration of 1 mg/l and almost 40,000 school children, mainly in deprived areas where the water is not fluoridated, receive fluoridated milk at a dose of 0.5 mg/189 ml milk in order to reduce the prevalence of dental caries.

Although low levels of F have an important role in prevention of dental caries, disturbances of enamel development (dental/enamel fluorosis) and bone homeostasis (skeletal fluorosis) can result from excessive retention of F in the body during tooth and bone development. Several factors are known to impact F metabolism and subsequently its retention in the body. An understanding of all aspects of F metabolism is critical to identify the biological effects of F and avoid F toxicity in humans.

Almost one-quarter of ingested F is rapidly absorbed from the stomach as hydrogen-fluoride and most of the remainder is absorbed more slowly from the proximal small intestine^2^. Plasma F concentration reaches its peak 30–60 min after F ingestion and returns to pre-ingestion levels during the next few hours depending on the F dose^3^. Approximately half of the absorbed F, in healthy adults and under normal conditions, binds to calcified tissues as flurohydroxyapatite while the remainder is excreted in urine. However, there is a considerable variation in the renal clearance of F among individuals which is dependent on a number of physiological and environmental factors. It has been suggested that, after entering the renal tubules, 10–90% of ionic F might be reabsorbed and returned to systemic circulation^2^. Since the mechanism of renal tubular reabsorption of F is pH-dependent, factors influencing acid-base status and urinary pH including certain drugs, high altitude, some respiratory diseases, metabolic diseases and physical activity can affect urinary F excretion and therefore F retention^2^,^3^.

It has been suggested^4^ that the current UK milk fluoridation scheme does not provide adequate protection against dental caries in children. Since fluoridated milk is given to British children on school days at their mid-morning break before going to the playground, the intensity of any exercise undertaken in the post-ingestion period, a potentially important factor in nutrient metabolism, might impact the ability of the body to retain F. No study on the effect of exercise on F pharmacokinetics has been undertaken in humans and there is very limited evidence on the effect of exercise on F metabolism in laboratory animals. Two studies with rats, which were exposed to an exercise regime of 0.3 m/sec and 2.25 m/min respectively for 1 h on a treadmill, reported 58–76% higher F levels in plasma of non-exercised rats compared with exercised rats^3^,^5^,^6^. The study by Lombarte *et al.* (2013)^5^, in which rats exercised for 30 days for 60 min at low intensity found a significantly higher F content of bones in the exercised rats. Although the effect of exercise on F metabolism was not directly investigated in two other studies with rats exposed to high NaF (600 ppm), through drinking water for one month^7^,^8^, an improvement in antioxidant status of the animals by the combined effect of temperature (25 °C and 30 °C) and exercise (swimming; 45 min/day for 10 days) was reported.

It is well known that exercise benefits bone health in children^9^ and adults^10^. Exercise is associated with reduced expression of osteocytes and increased expression of osteoblasts^11^. F is also one of only a few known agents that can stimulate osteoblast proliferation^1^. However, F demonstrates biphasic dose relationships being stimulatory to the precursors of osteoblasts at low doses and inhibitory to osteoclasts at high doses^12^.

Given the multiple health benefits of exercise^13^, several policy and environmental interventions have been implemented worldwide to promote and increase physical activity in communities^14^. However, very limited evidence and only from animal studies, is available on the effect of exercise on F metabolism. Therefore, in order to understand its effects in humans, this pilot study aimed to evaluate urinary F excretion (UFE) and plasma F concentration in young adults undergoing acute exercise with different intensities following ingestion of a 1 mgF tablet (2.2 mg NaF).

## Methods

### Ethical approval

This study was conducted according to the guidelines laid down in the Declaration of Helsinki and all procedures involving human subjects were approved by Teesside University, School of Health and Social Care Research and Governance Ethics Committee (protocol number: 154/13). Written informed consent was obtained from all participants prior to the experiment.

### Experimental Design

This pilot study was a human experiment with a randomised cross-over design, comparing observations within individuals. The sample size for this pilot study was considered based on the early animal studies by Whitford (n = 8)^3^ and the recent study by Lombarte *et al.* (n = 10)^5^.

### Participants

Ten healthy male and female adults, aged between 20–35y, volunteered to take part in the study. Inclusion criteria were: being resident in a non-fluoridated water area (<0.3 ppmF) for more than a year, as well as being moderately active and fit enough to take part in the prescribed exercise. Physical activity level and contra-indications to participate in physical activity were assessed respectively using the International Physical Activity Questionnaire^15^ and a Physical Activity Readiness Questionnaire^16^.

### Pre-experimental Procedures

After obtaining written informed consent, participants were asked to attend a pre-experimental session, one week before the trial, when background nocturnal urine and fasted blood samples (Background/pre-washout samples) were collected. At this session, the power output of the cycle ergometer (PowerTap Cycleops400, USA) was determined based on each participant’s Rate of Perceived Exertion (RPE) according to the Category Ratio Scale of Perceived Exertion (CR-10 RPE scale)^17^. The exercise intensities were set as: light intensity (RPE = 3), moderate intensity (RPE = 5), and vigorous intensity (RPE = 7). Heart rate was monitored during all exercise sessions with a heart rate monitor (Polar FF5, Polar Electro Oy, Finland) and recorded every minute during exercise.

F-free toothpastes were given to participants and they were asked to avoid using any F products for a one week wash-out period before as well as during the experimental period. They were also asked to avoid drinking tea, beer and tap-water (if leaving their stated residential area) and eating seafood during the washout and experimental periods. The subjects were asked to refrain from performing exercise other than habitual walking 48 h prior to and during experimental sessions.

### Experimental procedure and sample collection

**Figure 1:**
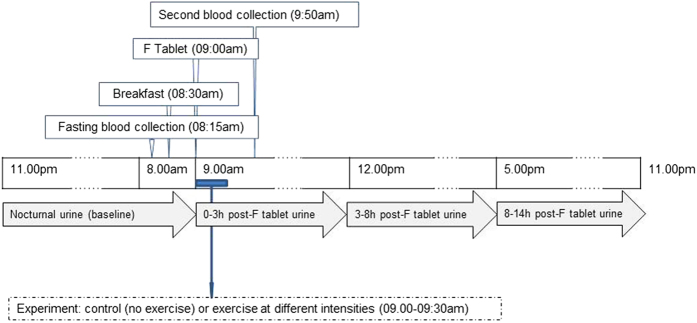
Experimental procedure and sample collection. After the one-week washout period, each participant underwent four randomly allocated experimental sessions including one no-exercise session and three exercise sessions at different intensities (light, moderate and vigorous) with approximately a one week interval between sessions.

After the one-week washout period, each participant underwent four randomly allocated experimental sessions including one no-exercise session and three exercise sessions at different intensities (light, moderate and vigorous) with approximately a one week interval between sessions (Fig. 1).

All sessions were conducted in an exercise laboratory at the same time of day. Exercise sessions consisted of a 5 min, self-selected speed warm up followed by 20 min of exercise on a stationary exercise cycle ergometer at the allocated intensity.

On each experimental session, a baseline venous blood sample (5 ml) was collected from each participant after an overnight fast. Participants were then provided with a low-F breakfast (<10 μgF) which comprised a cereal bar, a banana and 200 ml orange squash prepared with non-fluoridated bottled water (1:10v/v). The same food and drink items were consumed at the same time in each of the four sessions by all participants. After 30 min, at approximately 09:00am, participants were given a 1 mgF tablet (Fluor-a-day 2.2 mg NaF, Dental Health Products Ltd, UK) to swallow. The exercise session took from 09:00 to 09:30am. A second venous blood sample was collected, 50 min after ingestion of the F tablet, at about 09:50am.

Urine samples were collected by spontaneous voiding over a 24 h period during the following time periods:

1. One nocturnal pooled urine sample from midnight before the experimental session until 09.00am (baseline, pre-F tablet/pre-exercise);
2. One pooled urine sample from 09.00am to 12.00pm during the experimental session (0–3 h post-F tablet);
3. One pooled urine sample from 12.00pm to 17.00pm during the experimental session (3–8 h post-F tablet); and
4. One pooled sample from 17.00pm through to just before bed-time (~23.00pm) on the experimental day (8–14 h post-F tablet).

### Analytical Procedure

Urinary F concentration (μg/ml) was measured directly after adding total ionic strength adjustment buffer III (Orion Research) to standards and samples using a F-ion-selective electrode (F ISE). F concentrations in plasma (ng/ml) and breakfast items (μg/g) were measured, in triplicate, using a F-ion-selective electrode (Model 9609:Orion Research) coupled to a potentiometer (Model 720A) by a hexamethyldisiloxane (HMDS)-facilitated diffusion method^18^,^19^. This method, which was developed based on Taves method^19^ through an international collaborative project, has been previously reported in detail^18^. In summary, 1 ml H_2_SO_4_ saturated with HMDS was added to 1 ml sample (and standards) in a petri-dish and left at room temperature to diffuse overnight. The released F was trapped in an alkaline solution (50 μl of NaOH (0.05N), which was placed as 5 drops on the inside of the dish lid). After a minimum of 16 h diffusion, the lid was removed and 20 μl acetic acid (0.20N) was added and combined with the NaOH into a single drop of 75 μl. The F-ISE electrode was then placed in contact with the 75 μl solution and the mV reading recorded. A calibration curve was used to calculate F concentration of the sample.

Routinely, the standard operating procedure includes using a Standard Reference Material (SRM) to check the validity of the F analytical method. To the best of our knowledge, there is no SRM for plasma. However, the SRM 2668 (Toxic Elements in Frozen Human Urine), produced by the National Institute of Standards & Technology (NIST, Gaithersburg, MD, US) has been used as a quality assurance measure. The SRM 2668 comprises human urine at two levels of F (Level I and Level II) with a mean (SD) F concentration of 12.25 (0.14) and 18.83 (0.92) ppmF, respectively. The results for both levels have always been within the range (“mean – SD” and “mean + SD”) of F concentrations noted in the NIST certificate.

In addition, the reliability of the methods used was specifically confirmed by re-analysis of a minimum 10% of samples. In each session, 10% of samples was randomly selected for re-analysis, however, the re-analysis experiment was not performed on the same day as when the original samples were analysed in order to confirm between-day reproducibility. In total, 153 urine samples [(9 pre-washout samples @ one pre-washout sample/participant) + (144 post-washout/experimental samples @ 4 samples/session/participant x 4 sessions/participant x 9 participants)] were collected of which 16 were randomly selected for re-analysis. The total number of collected plasma samples was 81 [(9 pre-washout samples @ one pre-washout sample/participant) + (72 post-washout/experimental samples @ 2 samples/session/participant x 4 sessions/participant x 9 participants) of which 11 were randomly selected for reanalysis. All sample analysis and re-analysis was conducted in triplicate.

### Data analysis

Age-predicted maximal heart rate (HR) was estimated using the Tanaka *et al.* equation (208−0.7 × age)^20^. Percentage of heart rate maximum (%HRmax) was estimated from the average heart rate of each participant at different exercise intensities.

For each participant, urinary F excretion (UFE) in each individual time-controlled urine sample (μg) was quantified by multiplying the concentration of F (μg/ml) in each sample by its corresponding volume (ml). The Baseline UFE was subtracted from the UFE of each sample to derive the ‘Baseline-adjusted’ UFE. Total post-F tablet (Periods 2–4 inclusive: 14 h) UFE was calculated by summing the amount of F excreted in urine for the periods during and after each experimental session for each participant. Since urinary excretion of F returns to almost baseline values within the first 8 h after consumption of a F supplement (Ekstrand *et al.* 1994), the 8 h UFE was also calculated by summing the amount of F excreted in individual urine samples collected from 09.00am to 17.00pm (during periods 2 and 3). The UFE rate (μg/h) for each individual time-controlled urine sample was calculated by dividing UFE (μg) by the duration of the corresponding collection period (h).

The baseline plasma F concentration was subtracted from the F concentration in each plasma sample to derive the ‘Baseline-adjusted’ plasma F concentration.

The rate of F renal clearance (ml/min) for each individual at baseline (pre-) and 0–3 h post-F tablet was calculated by dividing rate of UFE per minute by plasma F concentration. The ‘Baseline-adjusted’ F renal clearance was then determined by subtracting post-exercise F renal clearance from baseline F renal clearance.

### Statistical Analysis

Data analyses were conducted using SPSS (version 21). Paired t-tests were applied to compare Background (pre-experimental) and Baseline nocturnal urine volumes and UFEs as well as fasted plasma F concentrations. The mean difference and 95% confidence interval (CI) for fasting plasma F concentration, urine volume and UFE between Background (pre-washout) and Baseline (pre-exercise) are presented.

However, due to the study being a pilot experiment to inform any subsequent definitive trial, only descriptive comparisons between control (no-exercise) and treatment (3 exercise intensities) were undertaken and the data are presented as mean and standard deviations.

## Results

Nine participants (4 males and 5 females) completed all aspects of the study. The mean (SD) age, weight, height and body mass index (BMI) of all participants was 24.7 (3.5) y, 67.7 (13.5) kg, 168.6 (11.3) cm and 24 (3) kg/m^2^, respectively (Table 1).

### Accuracy of the analytical method

The accuracy of the analytical method was confirmed by comparing the analysis and re-analysis measurements. The results showed no statistically significant differences between the two measurements with a mean (SD) difference of 0.009 (0.033) mgF/l for urine samples (n = 16) and 1.62 (2.56) ngF/ml for plasma samples (n = 11). The correlation between the analysis and re-analysis measurements was r = 0.997 (p < 0.001) for urine and r = 0.978 (p < 0.001) for plasma samples.

### Comparison of Background (pre-experimental) and Baseline data

The mean (SD) Background (pre-washout) and Baseline (pre-exercise) fasting plasma F concentration, nocturnal urine volume, urinary F concentration and UFE are presented in Table 2. The fasting plasma F concentration was significantly (p < 0.001) higher at Background compared with Baseline. However, no significant between-week differences in Baseline fasting plasma F concentrations were observed. There were no significant differences in either nocturnal urine volume, or urinary F concentration or nocturnal UFE between Background and Baseline.

### Comparison of control and different exercise intensities

Mean (SD) workload on the cycle ergometer for the different exercise intensities was 85.4 (8.7), 113.2 (16.2) and 129.6 (21.2) Watts for light, moderate and vigorous intensities, respectively. The mean (SD) % HRmax at light, moderate and vigorous intensities was 67 (10), 76 (11), 82 (9)% respectively.

The mean (SD) UFE at different time periods during the 24 h experimental session for control and each type of exercise regime is presented in Table 3. No substantial differences in UFE were found between control and the three different exercise intensities for any individual- as well as pooled-time periods.

**Figure 2:**
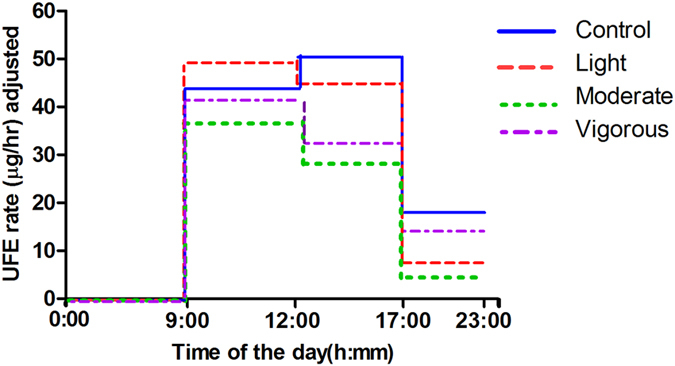
Mean Baseline-adjusted UFE rate (μg/h) across the time-controlled periods of collection according to exercise intensity. The mean Baseline-adjusted UFE rate tended to be higher for light intensity exercise over the first 3 h period after exercise, whereas it tended to be higher for the control conditions over the 3–8 h time controlled period. In general, moderate intensity exercise had a tendency to result in the lowest Baseline-adjusted UFE rate after exercise for all time controlled periods (0–3 h, 3–8 h and 8–14 h).

The mean Baseline-adjusted UFE rates across the time-controlled periods of collection are shown in Fig. 2. The mean Baseline-adjusted UFE rate tended to be higher for light intensity exercise over the first 3 h period after exercise, whereas it tended to be higher for the control conditions over the 3–8 h time controlled period. In general, moderate intensity exercise had a tendency to result in the lowest Baseline-adjusted UFE rate after exercise for all time controlled periods (0–3 h, 3–8 h and 8–14 h).

**Figure 3:**
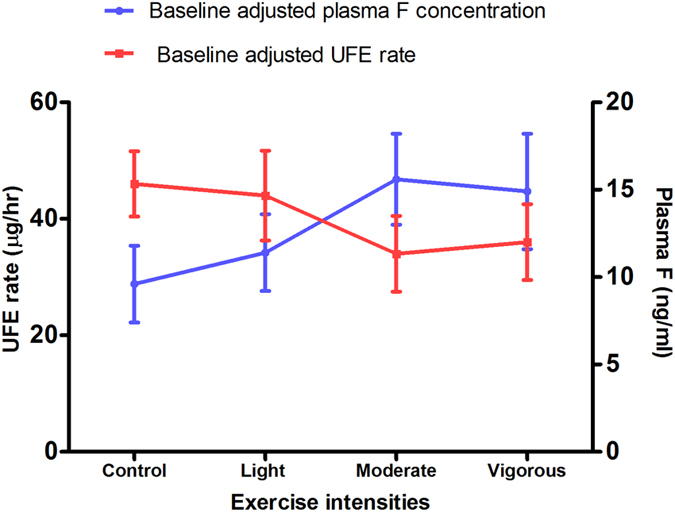
Mean (SE) Baseline-adjusted plasma F concentration (ng/ml) and mean (SE) Baseline-adjusted UFE rate (μg/h) over the 8 h post F ingestion period. Moderate and vigorous intensity exercise resulted in lower mean Baseline-adjusted UFE rates over the 8 h period in comparison with the control, whereas a higher plasma F concentration was observed for moderate and vigorous intensity exercise compared with the control.

The overall mean Baseline-adjusted UFE rate over the 8 h post F ingestion period and mean Baseline-adjusted fasting plasma F concentration are shown in Fig. 3. Moderate and vigorous intensity exercise resulted in lower mean Baseline-adjusted UFE rates over the 8 h period (34 and 36 μg/h, respectively) in comparison with the control (46 μg/h), whereas a higher plasma F concentration was found for moderate (15.6 ng/ml) and vigorous (14.9 ng/ml) intensity exercise compared with the control (9.6 ng/ml).

The mean (SD) Baseline-adjusted F renal clearance was 26.5 (9.0), 27.2 (30.4), 13.1 (20.4) and 18.3 (34.9) ml/min for no, light, moderate and vigorous exercise, respectively.

## Discussion

This study provides the first data on the effect of exercise with different intensities on plasma F concentration, renal F excretion and rate of F renal clearance in humans. The results suggests a rise in plasma F concentration and a decline in renal F excretion and clearance rates with increasing exercise intensity.

The study participants were young adults with a fairly narrow age range to minimise age-related variability in plasma F concentration between individuals (i.e. stage of skeletal development and rates of bone accretion and resorption). The present study showed a mean Background (pre-washout) fasting plasma F concentration of 62.0 ng/ml which was considerably higher than the corresponding figure of 19.3 ng/ml reported for a group of adults of similar age in the UK^21^. The high fasting plasma F concentration in the present study was mainly due to brushing with a fluoridated-toothpaste (1400 ppmF) by participants in the morning just before attending the pre-experimental session. However, there was a statistically significant drop in the mean fasting plasma F concentration (to 12.4 ng/ml) at Baseline (pre-exercise) which was primarily a result of switching to a non-fluoridated toothpaste during the washout and experimental period. The mean Baseline fasting plasma F concentration in the present study was consistent with the corresponding value of 10.5 ng/ml^22^, 12.7 ng/ml^3^, 12.4 ng/ml^23^ and 13.3 ng/ml^24^ reported for adults living in non-fluoridated areas.

Although daily UFE has been recognized as a biomarker for short-term F exposure, overnight fasting F concentration has been suggested as a useful indicator of chronic F exposure or potential bone F concentrations^25^. In the present study, no significant difference in the mean nocturnal UFE between Background and Baseline was found which can be explained by the dynamic relationship and fixed ratio between F concentrations in the extra-cellular fluid pools and the exchangeable pool in calcified tissues. When F intakes are very low, plasma F concentration declines and as a consequence F ion mobilizes from calcified tissues to extra-cellular fluid to maintain the fixed ratio of the F concentrations. This in turn results in a UFE at least as great as when F intakes are at a rather higher level^3^,^26^.

It has been suggested that plasma F concentration reaches its peak within 20–60 min following F ingestion^3^. However, in the present study the preferred timing of the second blood collection (i.e. 50 min after F ingestion) was based on more recent studies which have reported narrow ranges in peak plasma F concentration from 48 to 52 min in English adults (21–35y)^21^ and from 44 to 56 min in Brazilian adults (24–32y)^27^. Since UFE returns to baseline values in less than 8 h after consumption of a given F dose^26^, in the present study UFE over the 8 h following ingestion of the F tablet was determined and adjusted for baseline excretion to estimate the F excretion attributable to F dose.

In the present pilot study, the length of time for a ‘wash-out’ period was determined based on the previous studies in children and adults which have reported a rapid decrease in circulating F in the body of young children in the first 24 hours after discontinuation of F from water and toothpaste^28^, in contrast to a relatively constant UFE in adults being reached in one week following fluoridation of water^29^.

A trend in plasma F concentration, renal F excretion and rate of F renal clearance with an increase in exercise intensity was observed in this pilot study. The results showed a higher Baseline-adjusted plasma F concentrations following moderate (15.6 ng/ml) and vigorous (14.9 ng/ml) intensity exercise compared to light intensity (11.4 ng/ml) and no (9.6 ng/ml) exercise, implying a direct correlation between exercise intensity and plasma F level. A lower Baseline-adjusted UFE over the 8 h period as well as F renal clearance for the moderate and vigorous intensity exercise regimes compared with no- and light exercise (Fig. 3) suggest an increase in exercise intensity would decrease renal excretion of F.

In the present study, the exercise workloads and %HR max for the moderate and vigorous intensity groups were similar which could explain the similar results seen at these intensities. On the other hand the mean difference in exercise workload between the light and moderate exercise was quite noticeable (27.9 Watts), whereas the corresponding difference (16.4 Watts) between moderate and vigorous exercise was substantially lower. Similarly, the difference in exercise %HRmax between moderate and vigorous exercise was only 6%. It has been suggested heart rate should be 64 to 76% HRmax for moderate intensity exercise, while for vigorous intensity the range is 77 to 93% HRmax^30^. The average values in the present study were 76% for moderate and 82% for vigorous exercise, indicating a marginal difference between the two intensities. Future studies with exercise of a more vigorous nature (RPE>7; ∼90%HR max) may provide a better understanding of the physiological adaptations which occur at higher intensities.

In general, the present study found a large SD (i.e. variation) for renal clearance of F (e.g. mean (SD) for vigorous exercise: 18.3 (34.9) ml/min, indicating a wide variability among individuals at this intensity, while the variation was slightly less for light and moderate exercise 27.2(30.4) and 13.1(20.4), respectively. The literature also shows a wide variation in renal clearance of F from 12.4 to 71.4 ml/min^31^,^32^,^33^.

The pharmacokinetics of F can be influenced by physiological responses to exercise; however, the mechanisms by which exercise could affect F metabolism are not quite established. The renal clearance of F is directly associated with the pH in the renal tubules; a more alkaline urine is likely to increase F excretion. Therefore, the reported increase in urinary bicarbonate and pH with increasing exercise intensity^34^ implies a higher urinary F excretion. On the other hand, exercise increases sympathetic nervous system activity which might result in a 50% reduction in gastrointestinal secretion and blood flow^35^. This response might therefore reduce the rate and degree of gastrointestinal absorption of F, however some studies have reported no effect of exercise on gastric empting rate^35^. Production of lactic acid might push diffusion of F from extracellular to intracellular fluids and consequently a rise in the rate of F uptake by bone and other tissues which would therefore reduce plasma F concentration. Conversely, plasma F concentration may increase during exercise as a result of decreasing renal F excretion due to; a) vasoconstriction within the kidneys with reduction of renal blood flow and glomerular filtration rate due to increased sympathetic nervous system activity during exercise and; b) increased reabsorption of F from the renal tubules as a result of acidification of tubular fluid due to the lactic acid production during exercise^6^.

There are several reports on the effect of exercise at low intensity in rats but none in humans. In two similar but separate studies^3^,^6^, plasma F concentration was measured following either gastric intubation or intravenous administration of a single F dose of 5 mgF/kg in rats exposed to one week of light intensity (0.3 m/s) exercise on a treadmill for one hour. These two studies showed a significantly lower plasma F concentration in exercised rats (n = 8) compared with non-exercised rats (n = 8) with intra-gastric F dosing but no differences with intravenous F dosing. This suggested that the main effect of light exercise on F metabolism in rats was on the rate of gastrointestinal absorption of F. A recent study in which rats received 15 mgF/l in their drinking water for 30 days, also reported a significant reduction in plasma F concentration after running on a treadmill for 60 min at a light intensity for 30 days (n = 10) compared to a non-exercise group (n = 10)^5^.

In contrast with the animal studies, the present study with humans showed no effect of light exercise on plasma F concentration which could be due to differences in F dose and/or species (rat vs human). Although studies with an animal model allows easier control of the environment and a more in-depth analysis of F metabolism in different organs/tissues, there are some limitations in the use of animal models to reach appropriate conclusions regarding human F metabolism^36^. When rat animal models are used to study pharmacokinetics of F, it has been suggested that the F dose should be 4–5 times greater than that used in humans to achieve similar levels of F in plasma^37^. The water F concentration (15 mg/l) tested in the study by Lombarte *et al.* (2013)^5^ therefore corresponds to a water F concentration of 3 mg/l in humans. Studies looking at the effect of exercise might be better conducted in other animals which can perform moderate-to-heavy exercise rather than in rats which are reluctant runners. Among common laboratory animals, the canine model has been reported to provide more resemblance to the major features of human F pharmacokinetics^3^.

In conclusion, the observed rise in plasma F concentration and decline in renal clearance of F with increasing exercise intensity needs to be investigated in a larger trial. The findings of this pilot study could provide a robust estimate of the variability of the effect of exercise on F metabolism to inform sample size planning for any subsequent definitive trial.

## Additional Information

**How to cite this article**: Zohoori, F.V. *et al.* Effect of exercise on fluoride metabolism in adult humans: a pilot study. *Sci. Rep.* **5**, 16905; doi: 10.1038/srep16905 (2015).

## Figures and Tables

**Table 1:** Age, height, weight and body mass index (BMI) of individuals who participated in the study (n = 9).

Participant	Sex	Age	Height (cm)	Weight (kg)	BMI (kg/m^2^)
1	Female	22	163.6	72.3	27
2	Female	26	149.8	52.1	23
3	Male	24	168.3	70.4	25
4	Male	32	184.0	92.4	27
5	Male	23	184.5	80.3	24
6	Female	24	170.6	68.1	23
7	Male	21	174.5	52.7	17
8	Female	28	159.8	53.5	21
9	Female	22	162.0	67.5	26
Mean		24.7	168.6	67.7	24
SD		3.5	11.3	13.5	3

**Table 2:** Mean (SD) Background (pre-washout) and Baseline (pre-exercise) fasting plasma F concentration (Fasting F-plasma: ng/ml), nocturnal urine volume (ml), urinary F concentration (mg/l) and urinary F excretion (UFE: mg); and mean (95% Confidence Interval) differences between pre- and post-washout values.

Session	Fasting plasma-F (ng/ml)	Urine volume (ml)	Urinary F concentration (mg/l)	UFE (mg)
Background (pre-washout)	62.0 (20.3)	358 (102)	0.625 (0.347)	0.207 (0.084)
Baseline (post-washout, pre-exercise):				
Week 1	11.6 (2.1)	434 (224)	0.317 (0.259)	0.134 (0.072)
Week 2	18.0 (6.9)	389 (248)	0.458 (0.345)	0.176 (0.136)
Week 3	11.8 (5.1)	391 (168)	0.337 (0.172)	0.114 (0.037)
Week 4	9.7 (1.8)	438 (259)	0.351 (0.276)	0.141 (0.113)
Mean	12.8 (5.5)	414 (221)	0.377 (0.262)	0.142 (0.097)
Differences between Background and mean Baseline:				
Mean (95% Confidence Interval)	+49.3 (+34.1, +64.5)	−81 (−224, +62)	+0.217 (−0.011, +0.445)	+0.048 (−0.028, +0.125)
P value	<0.001	0.22	0.06	0.13

**Table 3:** Mean (SD) UFE (mg) for different time controlled periods during each 24 h experimental session for control and the three exercise intensities.

Session	Individual time period				Pooled time periods	
	Baseline^1^	0–3 h^2^	3–8 h^3^	8–14 h^4^	0–8 h^5^	0–14 h^6^
Control	0.123 (0.083)	0.294 (0.097)	0.229 (0.110)	0.157 (0.103)	0.502 (0.167)	0.703 (0.243)
Light exercise	0.154 (0.099)	0.330 (0.147)	0.198 (0.118)	0.184 (0.076)	0.529 (0.193)	0.808 (0.361)
Moderate exercise	0.145 (0.109)	0.267 (0.099)	0.178 (0.117)	0.154 (0.060)	0.424 (0.163)	0.697 (0.189)
Vigorous exercise	0.144 (0.108)	0.227 (0.103)	0.190 (0.094)	0.180 (0.099)	0.411 (0.170)	0.715 (0.279)

^1^Nocturnal pooled urine sample from midnight before the experimental session until 9 am on the experimental day before taking F tablet (baseline, pre-F tablet).

^2^Pooled urine sample from 09.00am to 12.00pm during the experimental session (0**–**3 h post-F tablet).

^3^Pooled urine sample from 12.00pm to 17.00pm during the experimental session (3**–**8 h post-F tablet).

^4^Pooled urine sample from 17.00pm through to just before bed-time (~23.00pm) on the experimental day (8–14 h post-F tablet).

^5^Combined 0–3 h and 3–8 h values.

^6^Combined 0–3 h, 3–8 h and 8–14 h values.
